# Prediction Model for Future Success of Early Orthopedic Treatment of Class III Malocclusion

**DOI:** 10.3390/children10020355

**Published:** 2023-02-10

**Authors:** Mi-So Lee, Van Nhat Thang Le, Jae-Gon Kim, Yeon-Mi Yang, Dae-Woo Lee

**Affiliations:** 1 Department of Pediatric Dentistry and Institute of Oral Bioscience, School of Dentistry, Jeonbuk National University, Jeonju 54896, Republic of Korea; 2 Research Institute of Clinical Medicine, Jeonbuk National University, Jeonju 54907, Republic of Korea; 3 Biomedical Research Institute, Jeonbuk National University Hospital, Jeonju 54907, Republic of Korea; 4 Faculty of Odonto-Stomatology, Hue University of Medicine and Pharmacy, Hue University, Hue 49120, Vietnam

**Keywords:** orthopedic treatment, class III malocclusion, prediction

## Abstract

This study aimed to identify predictors for successful post-treatment outcomes in early orthopedic class III malocclusion treatment with a facemask and hyrax expander appliance. The study was performed on lateral cephalograms from 37 patients at the start of treatment (T0), post-treatment (T1), and a minimum of three years after treatment (T2). The patients were grouped as stable or unstable according to the existence of a 2-mm overjet at T2. For statistical analysis, independent t-tests were used to compare the baseline characteristics and measurements of the two groups, considering a significance level of < 0.05. Thirty variables of pretreatment cephalograms were considered during logistic regression analysis to identify predictors. A discriminant equation was established using a stepwise method. The success rate and area under the curve were calculated, with AB to the mandibular plane, ANB, ODI, APDI, and A–B plane angles as predictors. The A–B plane angle was the most significantly different between the stable and unstable groups. In terms of the A–B plane angle, the success rate of early class III treatment with a facemask and hyrax expander appliance was 70.3%, and the area under the curve indicated a fair grade.

## 1. Introduction

Class III malocclusion is among the most challenging conditions to address with orthodontic treatment [1]. The prevalence of skeletal class III malocclusion varies widely among ethnic groups and different geographic regions. As reported in a previous review, prevalence rates are less than 8.3% in the United States, 3–5% in Brazil, 5% in the south of Italy, 2.8% in Germany, 2.3–14% in Japan, and 9–19% in South Korea [2]. These reports suggest a much higher rate of class III malocclusion among Asians than other ethnic groups.

The etiology of class III malocclusion is varied and complex, and this condition is associated with both genetic and environmental factors [3,4]. Moreover, several environmental factors can be involved in exacerbating class III malocclusion: oral habits, respiratory problems (enlarged tonsils and mouth breathing), incorrect posture, and congenital anatomic defects [5,6,7]. A previous study suggested that patients with unilateral cleft lip and palate exhibit delayed maxillary development similar to individuals with class III malocclusion [8]. Because of these many different factors, accurately predicting the possibility of success or failure after early orthopedic class III treatment is challenging, but it is necessary to facilitate the selection of the appropriate treatment protocols for good long-term outcomes.

Previous studies only evaluated early treatment of class III malocclusion in mixed dentition for a short-term period. Although recent systematic reviews suggested that the early treatment of class III malocclusion is effective in the short term, there is a lack of evidence on long-term benefits [9,10]. Another systematic review predicted the success of early treatment of class III malocclusion with different appliances, such as a chin cup, a facemask, a combination of chin cup and facemask, a facemask in combination with rapid maxillary expansion, cervical headgear, and functional appliances. It concluded that the accurate prediction of the orthopedic treatment outcomes of class III malocclusion was dubious because of the quality of the papers reviewed [11]. In addition, because of the large variety of predictors and differences among the developed prediction models, they doubted the existence of a universal predictor of the outcome of treatment of class III malocclusions [11].

Several prediction models have been suggested for short-term or long-term stability after early orthopedic class III treatment. For each prediction model, a variety of cephalometric predictors have been reported, such as the AB-mandibular plane angle, Wits appraisal, articular angle, N-perpendicular to point A, length of mandibular ramus, angulation of cranial base, inclination of mandibular plane to cranial base, lower face height, gonial angle, the position of the condyle with reference to cranial base, ramal length, and mandibular length [12,13,14,15,16,17,18,19,20,21,22,23]. However, some prediction models have low prediction accuracy [12,21]. In fact, use of fewer predictors in discriminant analysis is more useful in clinical practice, because it simplifies the treatment prediction and minimizes measurement errors. This leads to a null hypothesis: a model with a universal predictor would be established to improve predictive accuracy in patients with early orthopedic class III treatment.

This retrospective study aimed to identify predictors of initial skeletal morphology in patients with class III malocclusion and to establish a novel model with a universal predictor for successful post-treatment outcomes of a facemask and hyrax expander appliance using discriminant analysis.

## 2. Materials and Methods

In this study, patients seen between April 2004 and April 2015 at the Department of Pediatric Dentistry, Jeonbuk National University Dental Hospital, were considered. The following inclusion criteria were applied: (1) skeletal class Ⅲ malocclusion in the primary and permanent dentition characterized by an anterior crossbite and a Wits appraisal of –2.0 mm or less; (2) lateral cephalograms available for pretreatment (T0), post-treatment (T1), and a minimum of three years after treatment (T2); and (3) orthopedic treatment with a facemask and hyrax expander appliance, including maxilla protraction by semi-rapid expansion (one-quarter turn every other day) and the wearing of a facemask for at least 14 h per day. In the T1–T2 period, the patients wore a class III activator as a retainer at night for one year. The exclusion criteria were the existence of craniofacial syndrome and the application of orthopedic treatment using other modalities. This study was conducted following approval by the Institutional Review Board of Jeonbuk National University Hospital (No. CUH 2019-05-012, approved 1 June 2019).

Initially, a sample of 171 patients with class III malocclusion in the primary and mixed dentition was included. After screening the inclusion and exclusion criteria, a final total of 37 patients (mean age: 6.6 years) was selected for this study. According to the existence of an overjet of 2 mm after the completion of treatment (T2), the subjects were divided into stable (overjet > 2 mm) and unstable (overjet < 2 mm) groups. The following clinical data were collected through a chart review: sex (male or female), family history (is there a family history of mandibular prognathism?), rhinitis (is there rhinitis?), mouth breathing (is there chronic oral ventilation?), adenoid and tonsil hypertrophy (are there enlarged tonsils and adenoids?), oral habits (are there bad oral habits?), and cooperation (good or bad).

**Figure 1:**
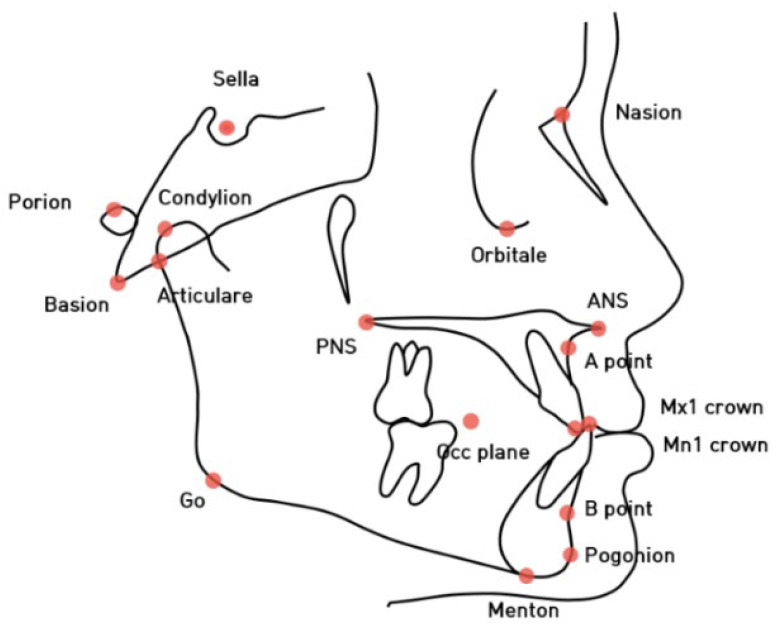
The cephalometric landmarks used in this study.

**Figure 2:**
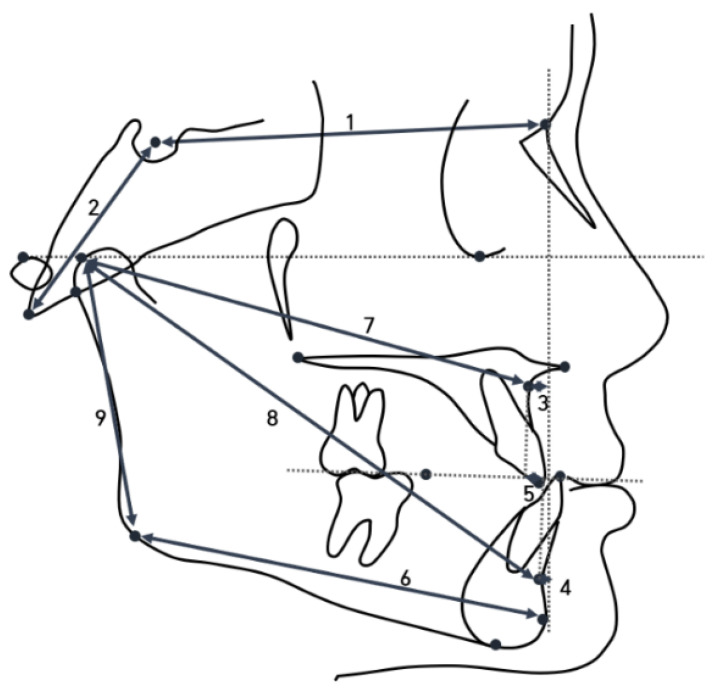
Linear measurements used in this study. 1, anterior cranial base; 2, posterior cranial base; 3, A point to N perpendicular; 4, pogonion to N perpendicular; 5, intermaxillary position to occlusal plane; 6, mandibular body length; 7, midfacial length; 8, mandibular length; and 9, mandibular ramus height.

**Figure 3:**
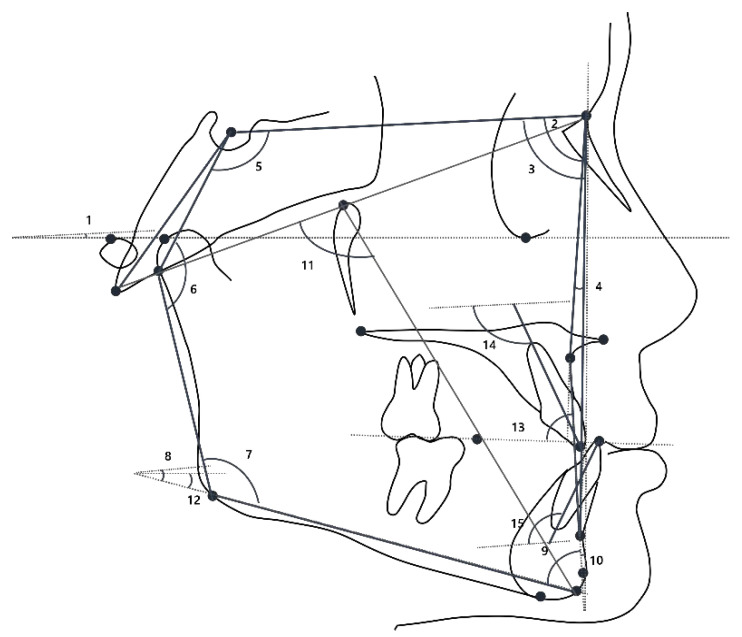
Angular measurements used in this study. 1, SN to the FH plane; 2, SNA angle; 3, SNB angle; 4, ANB angle; 5, saddle angle; 6, articular angle; 7, gonial angle; 8, SN-GoGn; 9, AB to the mandibular plane; 10, A–B plane angle; 11, facial axis; 12, mandibular plane angle; 13, A-B to the occlusal plane; 14, inclination of upper incisor; and 15, inclination of lower incisor.

Cephalometric analysis was performed at T0 and computed using V-Ceph software (version 6.0; Osstem, Seoul, South Korea), including 18 landmarks and 30 variables (linear and angular measurements). In this study, a third-year dental resident traced each lateral cephalogram. Before performing cephalometric analysis, the intra-rater reliability was evaluated. Eight lateral cephalograms were randomly selected from patients included in the study and were analyzed at two different times within one week. In both instances, the measurements obtained for each patient were analyzed through intraclass coefficient correlation (ICC). The ICC oscillated between 0.933 for the SNA angle and 0.991 for the SN to FH plane. These values indicated a high level of intra-rater concordance. Landmark positions and linear and angular measurements are presented in Figure 1, Figure 2 and Figure 3, respectively. The details of cephalometric landmarks and linear and angular measurements are described in Table 1. In addition, the analyses of lateral cephalograms were stratified according to six skeletal aspects: the cranial base (SN to FH, anterior cranial base, posterior cranial base, and saddle), maxilla (midfacial length), mandible (mandibular body length, mandibular ramus height, mandibular plane angle, mandibular length, and gonial angle), anteroposterior relationships (SNA, A point to N perpendicular, SNB, pogonion to N perpendicular, ANB, Wits, articular angle, and APDI), vertical relationships (sum, SN-GoGn, *Y*-axis to FH, ODI, AB to the mandibular plane, A–B plane angle, facial axis, FMA, and AB to the occlusal plane), and dental relationships (U1 to SN, L1 to NB, and IMPA).

### Statistical Analyses

All data were statistically analyzed using SPSS software (version 23.0, IBM, Chicago, CA, USA) with a significance level of *p* < 0.05. Independent t-tests were performed to compare baseline characteristics and cephalometric measurements between the stable and unstable groups. In addition, 30 variables of the lateral cephalograms at T0 were used in logistic regression analysis to identify key variables to distinguish between the stable and unstable groups. A stepwise method was applied to establish a discriminant equation with a universal variable. Using the discriminant equation, we stratified study participants into stable and unstable groups and calculated the success rate of prognosis. The success rate was based on accurate predictions of stable and unstable groups at T0 and T2.

For the evaluation of a predictive model, the area under the curve (AUC) was analyzed. In addition, the AUC value was classified as excellent (0.9–1.0), good (0.8–0.9), fair (0.7–0.8), poor (0.6–0.7), and failed (0.5–0.6) [24].

## 3. Results

### 3.1. Study Participant Characteristics

The mean age of the included patients at T0, T1, and T2 was 6.6 ± 1.7 years, 8.4 ± 1.8 years, and 13.7 ± 2.9 years, respectively, while the mean length of the treatment period (T0–T1) was 13.8 ± 5.2 months (Table 2). In addition, there were no significant differences between the stable and unstable groups in terms of sex, family history, rhinitis, mouth breathing, adenoid or tonsil hypertrophy, oral habits, or cooperation (Table 3).

### 3.2. Cephalometric Analysis

Differences between the stable and the unstable groups across the six skeletal aspects, including the cranial base, maxilla, mandible, vertical relationships, anteroposterior relationships, and dental relationships, were evaluated (Table 4).

The stable group had a larger ANB angle, smaller APDI angle, larger ODI angle, larger AB to mandibular plane angle, and smaller A–B plane angle compared with the unstable group (*p* < 0.05). Other variables were not significantly different (*p* > 0.05).

### 3.3. Discriminant and AUC Analysis

Five variables including AB to the mandibular plane, ANB, ODI, APDI, and A–B plane angles were used for discriminant analysis. In a stepwise manner, one variable suitable for distinguishing between the two groups (the most significant variable) was extracted, which was the A–B plane angle.

As shown in Table 5, unstandardized discriminant function coefficients of the selected predictive variable with a calculated constant established the following equation, which yields individual scores for assigning new patients to the stable or unstable group:

Individual score = 0.454 (A–B plane angle) − 0.013(1)

The critical score (mean value distinguishing between stable and unstable groups) was set to 0.000662. If a new patient with class III malocclusion showed that the individual score was lower than 0.000662, then the prognosis of early orthopedic treatment with a facemask and hyrax expander appliance was considered stable.

The success rate of the predictive model was 70.3%, as shown in Table 6. Regarding the predictability of treatment of skeletal class III malocclusion, the sensitivity and specificity values were 0.68 and 0.72, respectively. Furthermore, fair prediction performance (AUC of 0.724) of the discriminant function was achieved (Table 7).

## 4. Discussion

In this study, AB to the mandibular plane, ANB, ODI, APDI, and A–B plane angles were predictors of short-term prognosis in early orthopedic class III treatment. This suggested that the presence of increased vertical proportions and reduced overjet are indicators that early intervention might be less successful in the short term. A previous systematic review of 14 studies reported 35 cephalometric predictors of treatment outcome (20 linear, 13 angular, and two ratios) [11]. Apart from ODI, our predictors were reported in this review and in recent studies [12,25].

Among the five variables, the A–B plane angle showed the most significant difference between the stable and unstable groups and was the first variable to enter the stepwise discriminant model, suggesting that the A–B plane angle could be the best skeletal measurement for the prediction of class III early treatment. In addition, two previous studies established predictive models for Koreans [12,13], which were compared with our predictive model. Due to differences in subject age, follow-up duration, the method of early orthopedic treatment, and success criteria, the significant variables assigned to the equations were different among the studies.

The prediction model demonstrated moderate accuracy for the prognosis of early class III treatment in the short-term follow-up (70.3%). This discriminant will be helpful to clinicians and can be used to evaluate early orthopedic treatment, especially in skeletal Class III malocclusion in growing children. It may also help determine whether additional orthopedic treatment is needed before or during peak pubertal growth.

Although there are differences in patient characteristics, treatment protocols, success criteria, and evaluation time, the success rate of our study was higher than that reported in previous studies [12,13,15,16]. The success rate in some previous studies might have been lower because the treatment outcome was assessed after the confirmation of the completion of facial growth. There could have been late mandibular growth in some patients. Ghiz et al. collected data 3 years after treatment [16], Choi et al. collected data when their patients were approximately 19 years of age [12], and the authors of the remaining two studies collected data when their patients were approximately 17 years old [13,15]. In addition, our model had an AUC value of 0.724, which was a fair grade for prediction. The sensitivity and specificity values between our study and the previous investigations did not indicate a significant difference. Therefore, in comparison with other studies, our model demonstrated stability for predicting short-term outcomes in class III patients receiving early orthopedic treatment with a facemask and hyrax expander appliance.

Regarding early treatment protocols for class III malocclusion, facemasks and rapid maxillary expanders have been shown to be effective for enhancing maxillary growth and improving the overjet [26]. In a previous study, it was suggested that rapid maxillary expansion (two-quarter turns per day) and semi-rapid maxillary expansion (one-quarter turn every other day) have similar effects on dentofacial structure both in the transverse, vertical, and sagittal planes [27]. In combination with expansion therapy, a facemask is also used to improve the effectiveness of maxillary protraction. From a systematic review, clinicians suggested that a facemask should be used for 14–16 h a day [28]. In addition, a retainer with an activator is extremely important following orthopedic class III treatment to prevent relapse. Class III is not considered fully treated until growth is complete. Relapse is related to changes in dental tipping and maxillary rotation following the first month of facemask interruption [14,29,30].

Although our model with a universal predictor will help clinicians to simplify and improve prognosis prediction, some limitations influenced the success rate in this study. The first limitation of this retrospective study was that it was a short-term follow-up study conducted when the patient’s growth was not complete. Previous studies conducted in Korea showed that the mean age at T2 was 17.4 and 19.1 years, respectively [12,13]. However, the mean age at T2 was 13.7 ± 2.9 years in this study. The mean skeletal maturity index (SMI) for a 13-year-old male is SMI 6 [31], which means that skeletal growth is at the peak stage and therefore a remarkable amount of mandibular growth is expected. The patients who were classified into the stable group may eventually be classified into the unstable group in the long term. The final success of class III treatment should be evaluated after the completion of craniofacial growth.

The second limitation of this study was that the criteria used for determining the stable or unstable outcomes of treatment may be ambiguous. In our study, successful treatment was defined based on a 2-mm overjet at T2, for a total follow-up period of at least 3 years. However, overcorrection should be performed at T1 due to considering the relapse of anterior crossbite. Many clinicians recommended that the criterion for the successful orthopedic treatment of class III malocclusion should be more than 2 mm [32]. In previous studies, researchers used various criteria from 0 mm to 2 mm at T2 [12,16,18,21,33]. Since various criteria are observed in many class III malocclusion patients undergoing early orthodontic treatment, future studies will be needed to define clinical treatment outcomes.

Third, only hard tissue measurements were analyzed in this study. In further studies, it will be necessary to consider soft tissue measurements that may predict the treatment outcomes of skeletal class III cases. Finally, although we extracted information on major risk factors related to class III malocclusion, such as family history, oral habits, and patient medical history, we included only cephalometric variables in the analysis. We aimed to compare these findings with existing studies that created prediction models using only cephalometric variables. The performance of the predictive model should be improved by including important clinical information. Moreover, to achieve a higher performance for predicting the long-term outcomes of early orthopedic class III treatment, advanced methods such as machine learning or deep learning should be employed.

## 5. Conclusions

In early class III malocclusion treatment with a facemask and hyrax expander appliance, AB to the mandibular plane, ANB, ODI, APDI, and A–B plane angles were predictors of post-treatment outcomes. Furthermore, our prediction model using the A–B plane angle showed quite high accuracy. This model might be helpful for clinicians in terms of prognosis prediction and treatment decision making for growing patients with skeletal class Ⅲ malocclusion.

## Figures and Tables

**Table 1:** Descriptions of cephalometric landmarks, linear, and angular measurements.

Cephalometric Landmark	Description
Sella	Midpoint of the sella turcica or the hypophyseal/pituitary fossa
Nasion	Most anterior point on the frontonasal suture in the middle
Porion	Highest point on the roof of the left external auditory meatus
Orbitale	Lowest point on the inferior margin of the orbit, midpoint between the right and left images
Condylion (Cd)	Most superior point of the mandibular condyle (bilateral)
Basion (Ba)	Point at the center of the anterior border of the foramen magnum at the base of the occipital bone
Articulare (Ar)	Intersection of the dorsal contours of the processus articularis mandibulae and os temporale
Anterior Nasal Spine (ANS)	Tip of the bony anterior nasal spine in the midline or median plane
Posterior Nasal Spine (PNS)	Intersection of the continuation of the anterior wall of the pterygopalatine fossa and the nasal floor
A-point	Deepest point on the curved bony outline between the anterior nasal spine and prosthion
Maxilla 1 crown (Mx1 crown)	Tip of the maxillary incisor crown
Mandible 1 crown (Mn1 crown)	Tip of the mandibular incisor crown
Gonion (Go)	Mediolateral midpoint on the posterior most border of each gonial angle (the gonion is a bilateral structure)
B-point	Deepest midline point on the mandible between the infradentale and pogonion
Pogonion (Pog)	Most anterior point on the symphysis of the mandible
Menton (Me)	Lowermost point on the chin contour
**Linear cephalometric measurements**	**Description**
Anterior cranial base (N-S)	Length between the sella turcica and nasion
Posterior cranial base (S-Ba)	Length between the sella turcica and basion
A point to N perpendicular (A-Nperp)	Distance between point A and a line drawn perpendicular to the Frankfort plane (porion-orbitale) from point N
Pogonion to N perpendicular (Pog-Nperp)	Distance between pogonion point and a line drawn perpendicular to the Frankfort plane from point N
Intermaxillary position to occlusal plane (Wits appraisal)	Perpendicular distance between A- and B-points on the occlusal plane
Mandibular body length (Pog-Go)	Length between the pogonion and gonion
Midfacial length (Cd-A)	Length between the condylion and A-point
Mandibular length (Cd-B)	Length between the condylion and B-point
Mandibular ramus height (Cd-Go)	Length between the condylion and gonion
**Angular cephalometric measurements**	**Description**
SN to the FH plane	Angle between the Frankfort horizontal plane and sella-nasion plane
Position of maxilla (SNA angle)	Angle between the sella, nasion, and A-point
Position of mandible (SNB angle)	Angle between the sella, nasion, and B-point
Intermaxillary position (ANB angle)	Angle between the A-point, nasion, and B-point
Saddle angle (N-S-Ar)	Angle between the nasion, sella, and articulare
Articular angle (S-Ar-Go)	Angle between the sella, articulare, and gonion
Gonial angle (Ar-Go-Me)	Angle between the articulare, gonion, and menton
SN to Go-Gn	Angle formed by sella-nasion and gonion-gnathion lines (gnathion—the deepest point on the chin)
AB to the mandibular plane (A-B to Go-Gn)	Angle formed by point A-point B and gonion-gnathion lines
A–B plane angle (A-B to N-Pog)	Angle formed by point A-point B and nasion-pogonion lines
Facial axis (Pt-Gn to N-Ba)	Angle formed by pterygoid-gnathion and nasion-basion lines (pterygoid—eleven o’clock position from the pterygomaxillary fissure)
Mandibular plane angle (FH to Go-Gn)	Angle formed by the Frankfort plane and gonion-gnathion line
A-B to occlusal plane	Angle formed by the point A-point B line and the occlusal plane
Inclination of upper incisor (Mx1 crown to SN)	Angle between the maxilla 1 crown, sella, and nasion
Inclination of lower incisor (Mn1 crown to N-B)	Angle between the mandible 1 crown, nasion, and basion

**Table 2:** Demographic characteristics of the study participants.

		Unstable (*n* = 19)	Stable (*n* = 18)	Total (*n* = 37)
**Age (years)**	**T0**	6.9 ± 1.8	6.3 ± 1.8	6.6 ± 1.7
	**T1**	8.7 ± 1.9	8.1 ± 1.8	8.4 ± 1.8
	**T2**	13.8 ± 3.3	13.5 ± 2.5	13.7 ± 2.9
**Treatment period (T0–T1) (months)**		14.5 ± 1.8	13.1 ± 5.0	13.8 ± 5.2

**Table 3:** Comparison of baseline characteristics between the stable and unstable groups.

		Unstable (*n* = 19)	Stable (*n* = 18)	*p* Value
**Sex**	Male	7	12	0.070
	Female	12	6	
**Family history**	Y	8	7	0.842
	N	11	11	
**Rhinitis**	Yes	14	13	0.920
	No	5	5	
**Mouth breathing**	Yes	14	13	0.920
	No	5	5	
**Adenoid/tonsil hypertrophy**	Yes	13	14	0.522
	No	6	4	
**Oral habits**	Yes	13	11	0.642
	No	6	7	
**Cooperation**	Good	14	15	0.476
	Bad	5	3	

**Table 4:** Comparison of the two study groups in the initial stage.

Measurements	Stable (*n* = 18)	Unstable (*n* = 19)	Total (*n* = 37)	*p* Value
**Cranial base**				
SN to FH	8.19	8.43	8.31	0.808
Anterior cranial base	57.79	57.30	57.53	0.589
Posterior cranial base	28.38	27.53	27.94	0.385
Saddle	124.84	123.51	124.15	0.438
**Maxilla**				
Midfacial length	69.58	68.29	68.92	0.318
**Mandible**				
Mandibular body length	57.72	58.58	58.16	0.587
Mandibular ramus height	36.88	36.16	36.51	0.472
Mandibular plane angle	29.13	28.88	29.00	0.871
Mandibular length	92.45	92.32	92.38	0.942
Gonial angle	127.04	126.76	126.89	0.871
**Anteroposterior relationships**				
SNA	78.74	78.51	78.62	0.820
A point to N perpendicular	−2.58	−2.52	−2.55	0.948
SNB	77.64	78.72	78.20	0.279
Pogonion to N perpendicular	−7.51	−5.64	−6.55	0.249
ANB	1.10	−0.21	0.43	0.010 *
Wits	−7.83	−7.55	−7.69	0.868
Articular angle	145.49	147.08	146.30	0.507
APDI	85.11	87.49	86.33	0.039 *
**Vertical relationships**				
Sum	397.36	397.35	397.35	0.993
SN-GoGn	37.33	37.31	37.32	0.990
*Y*-axis to FH	62.30	61.29	61.78	0.405
ODI	67.27	63.62	65.39	0.011 *
AB to the mandibular plane	66.51	63.62	65.03	0.010 *
A−B plane angle	−0.99	1.00	0.03	0.009 *
Facial axis	85.56	86.80	86.20	0.308
FMA	29.13	28.88	68.92	0.871
AB to the occlusal plane	99.49	100.52	100.03	0.648
**Dental relationships**				
U1 to SN (angle)	95.93	98.51	97.26	0.444
L1 to NB (angle)	22.17	21.66	21.91	0.855
IMPA	87.20	85.62	86.39	0.576

* *p* < 0.05.

**Table 5:** Stepwise discriminant analysis.

Predictive Variables	Unstandardized Canonical Discriminant Function Coefficients
A–B plane angle	0.454
Constant	−0.013

**Table 6:** Classification results of discriminant analysis.

Group	Prediction		Total
	Unstable	Stable	
Unstable	13 (68.4%)	6 (31.6%)	19
Stable	5 (27.8%)	13 (72.2%)	18
**Success rate** of the prediction model: **70.3%**			

**Table 7:** The AUC values of the discriminant function.

AUC	Significance Level	95% Confidence Interval
0.724	0.020	0.560–0.887

## Data Availability

The datasets used and/or analyzed during the current study are available from the corresponding author on reasonable request.
